# Thai traditional massage increases biochemical markers of bone formation in postmenopausal women: a randomized crossover trial

**DOI:** 10.1186/1472-6882-13-69

**Published:** 2013-03-25

**Authors:** Sunee Saetung, La-or Chailurkit, Boonsong Ongphiphadhanakul

**Affiliations:** 1 Department of Medicine, Ramathibodi Hospital, Rama 6 Rd, Rajthevi, Bangkok, 10400, Thailand

**Keywords:** Bone formation marker, Postmenopausal women, Thai traditional massage

## Abstract

**Background:**

The effect of massage therapy on bone metabolism in adults has only scarcely been explored. In a randomized crossover trial, we investigated the skeletal effect of Thai traditional massage by examining the changes in biochemical markers of bone turnover.

**Methods:**

Forty-eight postmenopausal women participated in the study. All volunteers were randomized to a 2-hour session of Thai traditional massage twice a week for 4 weeks and a 4-week control period after a 2-week washout, or vice versa. Twenty-one subjects were allocated to receiving Thai traditional massage first, followed by the control period, while 27 were initially allocated to the control period.

**Results:**

Serum P1NP increased significantly after Thai traditional massage (*P* <0.01), while there was no change in serum osteocalcin or CTX. During the control period, there was no significant change in P1NP, osteocalcin or CTX compared to baseline. When age and height were taken into account, P1NP in postmenopausal women whose ages were in the middle and higher tertiles and whose heights were in the lower and middle tertiles (n = 22) had a 14.8 ± 3.3% increase in P1NP after massage (*P* <0.001), while no change in P1NP was found in the rest of the women (n = 26).

**Conclusions:**

Thai traditional massage results in an increase in bone formation as assessed by serum P1NP, particularly in postmenopausal women who are older and have a smaller body build. Future studies with larger samples and additional design features are warranted.

**Trial registration:**

ClinicalTrials.gov : NCT01627028

## Background

Mechanical loading favorably influences bone mass. Active exercise, as well as passive exercise through low-amplitude whole-body vibration, have been demonstrated to improve bone mass or delay bone loss [1,2]. Massage therapy has been shown to alleviate bone pain [3] and improve bone growth in both animals [4] and humans [5] during the postnatal period. However, the effect of massage therapy on bone metabolism in adults has only scarcely been explored.

Thai Traditional massage exerts pressure on the body in a rhythmic fashion. The massage performer uses the outstretched heels of both hands to exert pressure on the body of the subject approximately once every 1–2 seconds for 2 hours. It is likely that the physical load from Thai traditional massage may induce strain in the skeleton and affect bone, similar to other means of applying mechanical load. We have demonstrated in a previous study that Thai traditional massage results in an acute anabolic effect on bone, as assessed by biochemical markers of bone turnover [6]. It is unclear if a longer term of massage therapy would result in similar effects. Toward this end, a randomized crossover study was employed to investigate the skeletal effect of Thai traditional massage by examining the changes in biochemical markers of bone turnover.

## Methods

### Subjects

A randomized crossover design was used. A total of 48 postmenopausal women participated in the study. All were non-diabetic, as defined by a 2-hour plasma glucose level <200 mg/dL on a 75-g oral glucose tolerance test. Subjects having disorders that could affect bone metabolism – such as hyperparathyroidism, thyrotoxicosis, diabetes, rheumatoid arthritis and cancer, as well as those who were taking glucocorticoids or medications for osteoporosis – were excluded (Additional file 1). The study was approved by the Institutional Review Board of Ramathibodi Hospital. Signed informed consent was obtained from each subject prior to the study. All subjects were enrolled and the study performed at the Endocrine and Metabolism Unit of Ramathibodi Hospital, Bangkok from March 2011 to March 2012.

### Thai traditional massage

All volunteers were randomly assigned to either the treatment or the control group using a computer generated sequence. Subjects in the treatment group underwent a 2-hour session of Thai traditional massage twice a week for 4 weeks, while no intervention was given to subjects in the control group. After a 2-week washout period, subjects were switched to the other arm of intervention for 4 weeks.

Thai traditional massage was performed by a single masseuse throughout the study. Subjects were requested to change into comfortable, loose-fitting clothes and to lie flat on a firm mattress on the floor. The procedure consisted of the masseuse applying firm, rhythmic pressure over the volunteer’s body through the heels of her hands. The 2-hour procedure started with massaging the feet, and then the legs, arms, hands, back and neck, ending with a head massage (Table 1).

**Table 1:** Full body Thai traditional massage protocol*

**Subject position**	**Massage area**	**Duration (minutes)**
1. Lying on back	Feet- ankles	45
	Leg line and stretch	
	Abdomen, shoulders and arms	
	Hands	
	Neck, head and face	
2. Lying on side	Feet and legs	45
	Hip and buttock	
	Spine, waist and back	
	Shoulders, arms and hands	
3. Lying on chest	Feet- ankle	30
	Leg line and stretch	
	Buttock, hip, waist, and back	
	Shoulders	

* Consents were obtained from both subjects in the pictures.

### Biochemical measurement

Subjects were requested to refrain from exercise for 24 hours and to fast for at least 10 hours before blood was drawn in the morning. Blood samples were collected on the day of the initiation of the massage or control period, and on the day following each massage or control period. All samples were stored at −80 Celsius and analyzed in batch at the end of the study. Serum C-terminal telopeptide of type I collagen (CTx-I), total procollagen type 1 amino-terminal propeptide (P1NP), N-MID osteocalcin, and insulin were determined by electrochemiluminescence immunoassay on a Cobas e 411 analyzer (Roche Diagnostics, Mannheim, Germany). The intra-assay precision was 3.8%, 3.8%, 1.4% and 1.9%, respectively.

### Bone densitometry and measurement of body composition

Bone mineral density (BMD) and body composition were measured by dual-energy X-ray absorptiometry (DEXA) (Lunar Prodigy; GE Healthcare, Little Chalfont, UK). Daily calibration and quality control were performed regularly according to the manufacturer’s recommendations. Body composition and BMD of lumbar spine 1–4, femoral neck and total hip were measured in each subject.

### Statistical analysis

Changes in biochemical markers of bone turnover for both Thai traditional massage and control periods were assessed by paired Student’s *t*-test. Crossover statistical analysis was performed by the pkcross routine in Stata 12 software (StataCorp LP, College Station, TX), assuming no carryover effects. A *P*-value of less than 0.05 was considered statistically significant.

## Results

Table 2 demonstrates the clinical characteristics of the 48 study subjects. The mean age was 59.1 ± 4.3 years. All were postmenopausal women with an average of 8.9 ± 5.6 years since menopause. Seventeen (35.4%) were obese, based on BMI >25 kg/m^2^. Seven (14.6%) had osteoporosis either at the spine, femoral neck or total hip, according to a DEXA T-score of −2.5 or less at the corresponding sites.

**Table 2:** Characteristics of the study population

**Parameters**	**Mean** ± **SD(range) (n=48)**
Age (years)	59.1 ± 4.4 (49.8–66.6)
Years since menopause	8.9 ± 5.6 (1.2–24.3)
Body weight (kg)	57.1 ± 7.4 (40.0–73.8)
Height (cm)	153.1 ± 5.0 (141.5–163.5)
Body mass index (kg/m^2^)	24.3 ± 2.9 (18.1–30.9)

Twenty-one subjects were allocated to have Thai traditional massage first, followed by the control period, while 27 were initially allocated to the control period. When combined data from all subjects were analyzed, it was found that serum P1NP increased significantly after Thai traditional massage while there was no change in serum osteocalcin or CTX. During the control period, there was no significant change in P1NP, osteocalcin or CTX compared to baseline (Table 3). In a linear mixed model looking at the effect of massage and the sequence of intervention, it was found that Thai traditional massage significantly increased serum P1NP. No influence of the sequence of treatment allocation was found (Table 4).

**Table 3:** **Serum P1NP** (**median** (**range**)), **osteocalcin** (**mean** ± **SE**) **and CTX** (**median** (**range**)) **before and after massage or the control period**

	**Baseline**	**After massage**	***P***
**P1NP**	45.2 (40.2–50.3)	48.5 (44.2–53.3)	<0.01
**Osteocalcin**	22.8 ± 1.0	22.9 ± 0.8	NS
**CTX**	0.38 (0.33–0.43)	0.39 (0.34–0.43)	NS
	**Baseline**	**After control period**	***P***
**P1NP**	45.8 (41.9–50.1)	46.0 (42.0–50.4)	NS
**Osteocalcin**	23.0 ± 0.9	23.0 ± 0.9	NS
**CTX**	0.38 (0.34–0.43)	0.38 (0.34–0.43)	NS

Serum P1NP increased significantly after massage.

**Table 4:** Effects of treatment and its sequence on the change in P1NP after massage

	**MS**	**F**	***P***
**Sequence effect**	43.76	0.26	NS
**Treatment effect**	1294.95	5.69	<0.05

The effect of massage was statistically significant, while the sequence of treatment did not have a significant effect.

It is likely that body size as well as bone and fat mass may affect the responsiveness to the externally applied mechanical loading from Thai traditional massage. We investigated if there were associations between the percent change in P1NP after massage and age, body weight, height, and body composition. Table 5 demonstrates the change in serum P1NP according to body height tertiles. There was a significant increase in serum P1NP post-massage in subjects in both the lower and middle height tertiles. However, no change in serum P1NP was detected in subjects in the upper body height tertile. When age and body height were considered in combination, it was found that P1NP in postmenopausal women whose ages were in the middle and upper tertiles and whose heights were in the lower and middle tertiles (n = 22) had a 14.8 ± 3.3% increase in P1NP after massage (*P* <0.001), while no change in P1NP was found in the rest of the women (n = 26).

**Table 5:** **Changes in P1NP** (%) **after massage according to body height and age tertiles**

**Body height**	**Post**-**massage change in P1NP**	***P***
Tertile 1	+12.3 ± 3.9	<0.01
Tertile 2	+9.7 ± 4.0	<0.05
Tertile 3	+3.5 ± 3.5	NS
**Age**	**Post**-**massage change in P1NP**	***P***
Tertile 1	+3.9 ± 3.2	NS
Tertile 2	+13.6 ± 3.8	<0.01
Tertile 3	+8.1 ± 4.3	0.08

Serum P1NP increased significantly in the middle and lower body height tertiles. Likewise, serum P1NP increased significantly in the middle age tertile. The increase in P1NP in the upper age tertile almost reached statistical significance.

## Discussion

Massage has been widely utilized for the alleviation of a number of musculoskeletal disorders, including low back pain and bone pain from metastatic malignancy [3]. Despite its common utilization, studies showing evidence of the beneficial effects of massage therapy are still limited. Thai traditional massage has been shown to reduce pain and muscle tension in patients with scapulocostal syndrome [7]. Moreover, in a preliminary study on the acute effects of Thai traditional massage on biochemical markers of bone turnover, we demonstrated that a single 2-hour session can acutely increase serum P1NP, a marker of bone formation by 4.8% [6]. Using a randomized crossover design we demonstrated in the present study that two sessions per week of Thai traditional massage for 4 weeks resulted in a higher increase in P1NP, particularly in older individuals with smaller body built. The magnitude of change in P1NP from Thai traditional massage was comparable to the change in serum alkaline phosphatase in a study using vibration platform [8] but was much less than that achieved with parathyroid hormone, a potent bone formation agent used in the treatment of osteoporosis, which usually reaches 100% or higher [9]. Osteocalcin did not change significantly in the present study despite the changes in P1NP. Studies with bone forming agents such as parathyroid hormone and sclerostin monoclonal antibody have shown that osteocalcin is a less sensitive marker in response to treatments [10,11]. It is of note that the effect of Thai traditional massage on bone formation was more apparent in postmenopausal women of older age. While the improvement in the marker of bone formation cannot readily be extrapolated to an enhancement of bone mass or reduced fracture risk, our finding at least suggests that Thai traditional massage is likely to be beneficial to bone, particularly in women of advancing age among whom osteoporosis is a common health problem.

The effect of massage on bone metabolism in adults has scarcely been explored. However, a number of studies have investigated the influence of massage on the alteration of bone growth, particularly during the postnatal period. For example, massage in the early postnatal period was found to promote lean mass and bone growth in experimental animals [4]. In humans, when combined with physical activity, massage during the peri-neonatal period improves bone formation without changes in bone resorption [12]. Our findings are in keeping with those in infants, where massage therapy results in an increase in serum P1NP but not CTX.

It is well established that mechanical load affects bone cells. The strain characteristics that determine skeletal responses include strain magnitude [13], strain frequency [14] and strain rate [15]. There appears to be an inverse relationship between strain magnitude and frequency for inducing osteogenic effects. Low-magnitude mechanical load needs to be applied at high frequency in order to have an effect equivalent to high-magnitude mechanical load at lower frequency [16]. Moreover, the duration and cycle number of the loading are other factors related to the increase in osteoblast proliferation [17,18]. We showed in the present study that body height was inversely related to the increased in P1NP after Thai traditional massage. It is likely that this is partly because of tall postmenopausal women received less repetitive cyclic numbers of massage per body surface area than shorter women during the same period. In line with our previous study, we demonstrated in the present study that external periodic mechanical loading applied through Thai traditional massage is likely to have an anabolic effect on bone; this suggests that Thai traditional massage could be another option for enhancing bone health through mechanical loading.

Besides its direct effect on bone through mechanical loading, it is conceivable that Thai traditional massage may affect bone indirectly through the central nervous system. The adipokine leptin inhibits bone formation through a central nervous system delay [19]. Mechanical tactile stimulation reduces stress hormones and improves bone mineralization in rats [20]. Moreover, a study using functional MRI has shown that types of massage can influence brain cortical areas differently [21]. To what extent the effect of Thai traditional massage is due to its effect on stress hormones and the central nervous system is unknown. One of the other possible mechanisms involves ghrelin. It has been shown that ghrelin enhances bone formation [22], and that massage therapy in infants increases circulating ghrelin [23].

There are a number of limitations in the present study. The sample size is relatively small and may not be able to detect small effects on bone resorption. Moreover, although the current results are consistent with those of our previous study and of other studies in infants, it is still unclear if the increase in bone formation as reflected by bone markers will result in higher bone mass or reduced fractures. It is of note that only a single masseur performed the Thai traditional massage throughout the study. The result may not be readily generalized to other masseurs. Further studies, which include multiple practitioners, to confirm our results, as well as to investigate the effect of Thai traditional massage on bone mass or fractures, are warranted.

## Conclusions

The present study demonstrated that Thai traditional massage results in an increase in bone formation as assessed by serum P1NP, particularly in postmenopausal women who are older and have a smaller body build. Future studies with larger samples and additional design features are warranted.

## Competing interests

All authors have no competing interests.

## Authors’ contributions

BO conceived the idea of this study and drafted the manuscript. SS participated in its design and coordination and helped to draft the manuscript. LC performed the electrochemiluminescence immunoassay. All authors read and approved the final manuscript.

## Pre-publication history

The pre-publication history for this paper can be accessed here:

http://www.biomedcentral.com/1472-6882/13/69/prepub

## Supplementary Material

Additional file 1 CONSORT 2010 Flow Diagram. Click here for file
