# Supplementary material for: Thai traditional massage increases biochemical markers of bone formation in postmenopausal women: a randomized crossover trial
Source: BMC Complement Altern Med. 2013 Mar 25;13:69. doi: 10.1186/1472-6882-13-69 (PMC3770450; doi:10.1186/1472-6882-13-69)
Supplement: Additional file 1 — CONSORT 2010 Flow Diagram. [file 1472-6882-13-69-S1.doc]

**CONSORT 2010 Flow Diagram**

Assessed for eligibility (n= 101)

**Allocation**

**Analysis**

**Follow-Up**

**Enrollment**

Excluded (n= 29)

♦  Not meeting inclusion criteria (n= 29)

♦Analysed (n= 21)

♦Lost to follow-up because of lack of interest (n=1)

Allocated to massage/control (n= 36)

♦ Received allocated intervention (n= 22 )

♦ Did not receive allocated intervention because of unwillingness to participate (n=14)

♦Lost to follow-up because of lack of interest and transportation problem (n=3 )

Allocated to control/massage (n= 36)

♦ Received allocated intervention (n= 30 )

♦ Did not receive allocated intervention because of unwillingness to participate (n= 6)

♦Analysed (n= 27)

Randomized (n=72)
